# Comparative assessment of two frailty instruments for risk-stratification in elderly surgical patients: study protocol for a prospective cohort study

**DOI:** 10.1186/s12871-016-0276-0

**Published:** 2016-11-14

**Authors:** Daniel I. McIsaac, Monica Taljaard, Gregory L. Bryson, Paul E. Beaule, Sylvain Gagne, Gavin Hamilton, Emily Hladkowicz, Allen Huang, John Joanisse, Luke T. Lavallée, Hussein Moloo, Kednapa Thavorn, Carl van Walraven, Homer Yang, Alan J. Forster

**Affiliations:** 1 Department of Anesthesiology and Pain Medicine, University of Ottawa, Ottawa, Canada; 2 Ottawa Hospital Research Institute, Ottawa, Canada; 3 Department of Anesthesiology, The Ottawa Hospital, Ottawa, Canada; 4 School of Epidemiology, Public Health and Preventive Medicine, University of Ottawa, Ottawa, Canada; 5 University of Ottawa and The Ottawa Hospital, Ottawa, Canada; 6 Anesthesiology Resident, University of Ottawa, Ottawa, Canada; 7 Geriatric Medicine, The Ottawa Hospital, Ottawa, Canada; 8 Hôpital Montfort, Ottawa, Canada; 9 Division of Urology, University of Ottawa, Ottawa, Canada; 10 Division of General Surgery, University of Ottawa, Ottawa, Canada; 11 Institute for Clinical Evaluative Sciences, Toronto, Canada; 12 Medicine and Epidemiology, University of Ottawa, Ottawa, Canada; 13 Department of Medicine, University of Ottawa, Ottawa, Canada; 14 The Ottawa Hospital, Ottawa, Canada

**Keywords:** Geriatrics, Surgery, Prognosis, Disability, Patient reported outcome measures, Epidemiology, Frailty, Outcomes

## Abstract

**Background:**

Frailty is an aggregate expression of susceptibility to poor outcomes, owing to age-, and disease-related deficits that accumulate within multiple domains. Older patients who are frail before surgery are at an increased risk of morbidity and mortality, and use a disproportionately high amount of healthcare resources. While frailty is now a well-established risk factor for adverse postoperative outcomes, the perioperative literature lacks studies that: 1) compare the predictive accuracy of different frailty instruments; 2) consider the impact of frailty on patient-reported outcome measures; and 3) consider the acceptability and feasibility of using frailty instruments in clinical practice.

**Methods:**

We will conduct a multicenter prospective cohort study comparing the predictive accuracy of the modified Fried Index (mFI) with the Clinical Frailty Scale (CFS) among consenting patients aged 65 years and older having elective major non-cardiac surgery. The primary outcome will be disability free survival at 90 days after surgery, a patient-reported outcome measure. Secondary outcomes will include complications, length of stay, discharge disposition, readmission and total health system costs. We will compare the accuracy of frailty instruments using the relative true positive rate and relative false positive rate. These measures can be interpreted as the relative difference in the probability of one instrument identifying a true case of death or new disability compared to another instrument, or the relative difference in the probability of one instrument identifying a false case of death or new disability, respectively. We will also assess the acceptability and feasibility of each instrument.

**Discussion:**

Frailty is an important prognostic factor in the growing population of older patients having surgery. This study will provide novel findings regarding the choice of an accurate, clinically useable frailty instrument in predicting patient reported outcomes, as well as morbidity, mortality and resource use. These findings will inform current practice and future research.

## Background

Frailty is an aggregate expression of susceptibility to poor outcomes, that is due to age-related and disease-related deficits [1] which accumulate within physical, physiological, cognitive, and psychosocial domains [2]. Frailty-related risk is manifest through a vulnerability to stressors [3] that translates into increased risk of mortality and adverse health outcomes compared to non-frail individuals [2, 4, 5]. The prevalence of frailty increases exponentially with age; [6] approximately 10% of 65–75 year olds are frail while over 40% of individuals over 80 years of age are frail [5].

Patients 65 years of age and older comprise an ever-increasing proportion of the surgical population [7]. On its own, advanced age is an important predictor of adverse post-operative outcomes [8–10]. However, marked variations in outcomes exist between surgical patients of the same age [11]. Given the significant physiological stress induced by surgery, it is not surprising that frailty is a key factor explaining this variation. Frailty independently predicts mortality, morbidity, extended length of stay, and institutional discharge after surgery [12–18].

Identifying frail patients prior to surgery could improve the care and outcomes of older patients through a variety of mechanisms: identification of high risk patients can lead to improved decision making and informed procedural consent [19], while the multidimensional nature of frailty means that one or more of its underlying components may be amenable to optimization at the patient-level [20]. Furthermore, frail patients may benefit from optimization of the healthcare system, such as centralization of care.

Numerous frailty instruments exist to identify or diagnose individuals as frail [21]. Although different instruments identify closely related high-risk groups of patients, instruments are known to vary in their content validity, feasibility, and ability to predict adverse health outcomes [4]. To date, many different frailty instruments have been used to identify high-risk patients in the acute care setting [22]. However, few studies have compared the performance of different frailty instruments in identifying high-risk frail older patients having surgery. Furthermore, the majority of published studies describe the association of frailty with routinely measured postoperative outcomes, such as in-hospital or 30-day mortality, complications, and length of stay (LOS) [22]. While these outcomes provide important information for patients, clinicians and other stakeholders, the impact of frailty on postoperative patient-reported outcome measures (PROMs) is poorly described [22]. The association of preoperative frailty with postoperative PROMs is clearly needed to provide more patient-centered insights into the epidemiology of perioperative frailty.

Finally, despite recent recommendations from a variety of national speciality societies that frailty be a routine part of preoperative assessment in older patients [23, 24], there is currently no evidence that this recommendation is consistently applied. In addition to a lack of guidance regarding which frailty instrument to use in the perioperative setting, there is a lack of knowledge regarding the acceptability and feasibility of different frailty instruments in perioperative practice.

Based on the knowledge gaps described above, the objectives of this prospective cohort study are to 1) compare the accuracy of the modified Fried Index [1] (mFI) and the Clinical Frailty Scale [2] (CFS) in predicting disability free survival after elective non-cardiac surgery, 2) compare their acceptability and feasibility in clinical practice, and 3) explore the impact of adding other variables from guideline-recommended preoperative geriatric assessment to the discriminatory performance of frailty measures to predict disability free survival (DFS).

## Methods

### Study design and setting

We will conduct a prospective, multi-center cohort study at a multi-site academic health sciences center (The Ottawa Hospital (TOH)), and a community-practice focussed single site hospital (Hôpital Montfort). TOH is a 900-bed tertiary care center that is the regional referral center for trauma, vascular, neuro, thoracic and complex oncology surgery, and serves a catchment population of 1.2 million people. Hôpital Montfort is a 300-bed hospital with a community-focussed practice and serves a predominantly French-speaking population. Both hospitals are located in Ottawa, Canada. All patients will be recruited in the preoperative assessment unit of each hospital, where patients are seen by a registered nurse or physician anesthesiologist for medical assessment prior to surgery. Research ethics board (REB) approval has been granted by each study center.

### Study population

Individuals aged 65 years or older on the day of their elective, major non-cardiac surgery will be eligible to participate. Major non-cardiac surgery will be defined as surgery not involving the heart, pericardium, or cardiopulmonary bypass with an expected length of stay of at least 2 days, and will be limited to primary procedures (i.e., revisions of previous surgical procedures will not be included).

### Inclusion and exclusion criteria

All consenting patients having an eligible procedure who are able to communicate in English or French, and who are able to be contacted by telephone for follow up will be *included*. Because some frail patients may be cognitively impaired, REB approval includes the ability for informed study consent from a patient’s health decision maker. However, patients unable to answer outcome scales will be excluded. No other specific exclusion criteria will be applied.

### Primary independent variables

Our primary exposure of interest is the presence and severity of frailty as measured by two existing frailty instruments with evidence of predictive utility and feasibility in the acute care setting. The mFI [1] is a 5-point frailty instrument based on a frailty phenotype model which consists of measures of weight loss, grip strength, exhaustion, walking speed, and activity level. This instrument has been shown to predict adverse events and institutional discharge after surgery with moderate discrimination, and has been independently tested in several surgical populations [25, 26]. The mFI is currently recommended by the ACS/AGS best practice guidelines for frailty assessment before surgery [23]. The CFS is a 9-point global frailty scale based on clinical evaluation in the domains of mobility, energy, physical activity and function [2]. Despite its relative simplicity and ease of use, the CFS is equally discriminative in predicting mortality compared to frailty indices containing 40+ variables [4, 27]. Additionally, the CFS may be more feasible for clinical use than other frailty instruments based on a smaller number of missing responses [4]. In the acute care setting, increasing scores on the CFS are associated with increasing risk of in-hospital mortality [28–30]. All frailty assessments will be carried out by a study clinician or one of three trained clinical research assistants (CRA) during each patient’s preoperative assessment unit visit. These visits typically occur 1–4 weeks prior to surgery. Assessments performed by CRAs will be reviewed by a study clinician to ensure accuracy.

Frailty will be defined by typically used cut-offs for the mFI (≥3) and CFS (≥4), however, various alternative forms that account for the degree of frailty will also be tested (see *Analysis* section).

### Outcomes

The primary outcome will be disability free survival (DFS) at 90 days after surgery, measured as a binary indicator of being alive with a disability score of 25% or less, based on the 12-item World Health Organization Disability Assessment Schedule 2.0 (WHODAS) [31]. For people with a baseline WHODAS higher than 25%, DFS will be defined as alive with a disability level of no more than 8% above baseline [32]. The WHODAS will be administered by telephone by a CRA blinded to the participant’s preoperative frailty status. Previous studies confirm the performance and applicability of the WHODAS in a variety of disease states, including stroke [33], spinal cord injury [34], trauma [35], chronic disease states [36], and after surgery [32]. In addition to 90-day measurement, we will also assess DFS at 30 and 365 days after surgery. A 90-day primary outcome ascertainment point was chosen for two reasons. First, outcomes beyond the traditional 30-day window are increasingly seen as high-value metrics for patients [37], and second, the WHODAS assesses disability in the 30 days prior to questionnaire administration. Therefore, a 30-day postoperative WHODAS score may be highly influenced by the initial surgical insult, as opposed to each patient’s longer-term functional trajectory.

Secondary outcomes will include complications (defined by Post-Operative Morbidity Survey [38] criteria, applied through a post-discharge chart review), patient safety events (defined by *International Classification of Diseases Tenth Edition* codes grouped by patient safety indicator clusters using Type 2 diagnoses [39]), hospital LOS, total health system costs (using standard person-level cost algorithms to identify costs of the index hospitalization, and total costs up to 30, 90 and 365 days after surgery [40]), discharge disposition (home vs. supported or institutional discharge). All secondary outcomes other than complications will be ascertained using validated methods through linkage to provincial health administrative data.

### Covariates

Baseline clinical and demographic characteristics collected will include data reported by patients or personal support members/caregivers: age, gender, cognitive status (using the AD8 questionnaire [41]), depression (Patient Health Questionnaire-2 screener [42]), alcohol abuse (CAGE questionnaire [43]), self reported Elixhauser comorbidities [44], current smoking status, presence of hearing or visual deficits, recent unexpected weight loss, history of falls in the previous 12 months, ability to independently mobilize, dress, prepare meals, shop and take care of finances. Additionally, we will capture covariates from health administrative data: American Society of Anesthesiologists score, laboratory data ordered in the preoperative assessment unit, number of prescription medications, use of high-risk medications (opioids, benzodiazepines, anti-histamines, other anti-cholinergic drugs), anesthesia type, duration of surgery, surgery type (as defined by the first 5 digits of the Canadian Classification of Interventions code), and surgical risk (as defined by the Procedural Index for Mortality Risk [45]). Pertinent covariates were identified from existing preoperative risk prediction tools, as well as the ACS/AGS Optimal Preoperative Assessment of the Geriatric Surgery Patient Guideline.

### Acceptability and feasibility

The acceptability of each frailty instrument to clinicians and patients will be assessed using Likert scale scored questions based on relevant queries from previously validated acceptability scales [46]. We will recruit a convenience sample of 30 clinicians in the preoperative assessment unit who are willing to administer each frailty instrument in the clinical assessment of a participating patient. All patient participants will be asked to assess the acceptability of each tool and the full data collection process. Feasibility will be assessed in the domain of practicality, based on presence and frequency of missing items and time of administration [47]. Feasibility and acceptability scales are included in the Appendix.

### Descriptive analysis

Patient and clinical characteristics will be compared across frail and non-frail strata using descriptive statistics.

### Primary analysis

Our primary analysis will calculate the predictive accuracy of each of the CFS and the mFI in identifying patients who develop a new disability or die in the 90 days after surgery using concepts from diagnostic accuracy testing. Specifically, we will compare the true positive rate (i.e., sensitivity), as well as the false positive rate (i.e., 1-specificity) between instruments. The true positive rate reflects the benefit of screening for adverse outcome risk using a particular instrument by quantifying the proportion of people who will actually experience the adverse outcome and who are identified by the instrument. Alternatively, the false positive rate reflects the disadvantage of using the instrument by quantifying the proportion of people who are erroneously identified as likely to experience an adverse outcome [48]. We will use the standard dichotomization of each tool as the test positive definition for each instrument.

To compare the accuracy of the two frailty instruments head to head we will use the relative true positive rate (rTPR), and relative false positive rate (rFPR) with 95% confidence intervals [48]. The rTPR can be interpreted as the relative difference in the probability of one instrument identifying a true case of death or new disability (i.e., a true positive) compared to another instrument. For example, a rTPR of 1.2 would be interpreted as the CFS identifying 20% more cases of death or new disability than the mFI. The A rFPR of 3.0 would be interpreted as one test incorrectly identifying 3 times more people as likely to experience death or new disability. A rTPR or rFPR with a confidence interval excluding the null value (i.e., 1) will be considered to indicate a significant difference in accuracy between frailty instruments. Because different clinical scenarios may call for an instrument with higher sensitivity, while others may require higher specificity, the preferred instrument will ultimately be determined by the indication for its use. However, if one instrument is found to have a significantly higher true positive rate and a significantly lower false positive rate it would likely be superior in most settings.

We will also perform subgroup analyses in pertinent procedural cohorts, including total joint arthroplasty, intra-abdominal surgery, oncology, and vascular surgery. In each subgroup, a separate rTPR, rFPR and associated 95% CIs will be calculated.

### Secondary analyses

In addition to the dichotomized definition of frailty used in our primary analysis, both the mFI and CFS provide an ordinal frailty scale. Therefore, to assess the discriminatory performance of each frailty scale expressed on an ordinal basis, we will perform a receiver operator characteristic (ROC) curve analysis to identify whether a different optimal cut-point may exist for each frailty instrument in the perioperative setting [49]. If a cut-point is identified that is different than those typically used, we will repeat our rTPR analysis using the newly identified cut point.

We will also measure the strength of association between frailty and primary and secondary study outcomes using regression coefficients and 95% confidence intervals from an appropriate model: for dichotomous outcomes we will calculate the odds ratio using logistic regression, for length of stay (time to discharge) we will calculate the hazard ratio using Cox proportional hazards regression, for hospital and health system costs we will use relative difference from generalized linear regression.

### Exploratory analysis

In addition to frailty, the ACS/AGS Optimal Preoperative Assessment of the Geriatric Surgical Patient recommends assessment of a variety of patient conditions and characteristics [23]. Therefore, to explore the contribution of other variables to predicting death or new disability in our cohort of elderly patients, we will perform exploratory multivariable modelling by entering all clinically important preoperative covariates together with frailty status into a logistic regression model and using a backward elimination algorithm to produce a parsimonious model that can predict the occurrence of new death or disability. The discrimination of the model will be assessed using area under the ROC curve (C-statistic) with 95% confidence interval, and calibration will be assessed using the Hosmer-Lemeshow goodness of fit test.

### Assessment of acceptability and feasibility

We will measure the acceptability of each instrument for clinicians by reporting the mean and standard deviation of each acceptability question for each tool, as well as an aggregate mean and standard deviation across questions. For patients, we will calculate the mean and standard deviation for each instrument in relation to the perceived of ease of use, and whether the patient would be willing to complete the instrument again in the future.

We will measure the feasibility of each instrument by expressing the mean number of missing items from each frailty instrument. We will also calculate the mean time in seconds that were required to complete each instrument.

### Sample size

The sample size calculation is based on the primary objective of comparing the mFI (≥3) and CFS (≥4) with respect to their accuracy in identifying patients with events at 90 days after surgery. We used the sample size calculation approach described by Alonzo et al. for paired comparisons of diagnostic test accuracy [48]. The approach is based on the joint 95% confidence region around the relative True Positive Rate (rTPR) and False Positive Rates (rFPR), but since prevalence of events is expected to be low, the sample size is driven entirely by the calculation for rTPR. Although no measures of sensitivity for detecting true cases of DFS are available for the CFS or mFI, existing literature suggests that the mFI has a 61% sensitivity for identifying true cases of postoperative complications [16]. Therefore, we assumed a TPR for the mFI as a sensitivity of 0.6. We would consider a 30% relative difference in the TPR for the CFS to be clinically meaningful. We further assumed a concordance probability (i.e., the proportion of patients classified as positive by both tests, of 0.45). To have 80% power at a 5% significance level to detect a rTPR of 1.3, the required number of cases is 117. At a prevalence of events of 18%, this translates to a total required sample size for the cohort of 648 patients.

## Discussion

This multicenter prospective cohort study will address multiple knowledge gaps in the perioperative frailty evidence base. We will describe the comparative performance of two leading frailty instruments in predicting a patient reported outcome measure (DFS), as well as commonly reported postoperative outcomes. Furthermore, we will assess the predictive value of two frailty instruments in addition to known preoperative risk factors. Finally, we will provide new information regarding the acceptability and feasibility of the mFI and CFS, which in addition to their predictive accuracy, should guide clinicians in choosing an appropriate instrument with which to perform preoperative frailty assessments.

Given the rapid aging of Western populations [50, 51], and the increasing prevalence of older patients in the perioperative setting [7], accurate and routine identification of the high risk strata of frail older patients is needed to inform patient and surgical decision making, and to appropriately plan perioperative care. The plethora of available frailty instruments combined with a lack of comparative studies creates a substantial barrier for uptake of frailty instruments by clinicians [52], despite such assessments being recommended by best practice guidelines [23, 24]. Furthermore, the association of available frailty instruments with patient reported outcome measures, which have recently been identified as key areas for new research [53], is poorly studied. This lack of data may cloud the interpretation of frailty status before surgery for patients and clinicians. Our study will provide important insights into each of these needed areas of research.

Feasibility and acceptability are known external barriers to practice change [54]. Although some evidence exists regarding the feasibility of the mFI in perioperative practice and the CFS in non-surgical settings, the comparative feasibility and acceptability of different frailty instruments is not well described. Our study will supplement measures of comparative diagnostic accuracy between the CFS and mFI with acceptability and feasibility measures based on accepted frameworks. The combination of these data should influence the future practice of frailty assessments and the application of best practice guidelines.

## Appendix

Measures of feasibility and acceptability

### Acceptability

For clinicians: Was the frailty instrument… (1 being least easy/5 being most easy)

1. Easy to use? (1-2-3-4-5)
2. Useful in my practice? (1-2-3-4-5)
3. Beneficial to patients care? (1-2-3-4-5)
4. Miss important clinical factors? (1-2-3-4-5)
5. Difficult to use for environmental/logistical reasons? (1-2-3-4-5)

For patients: Was this questionnaire (or group of questionnaires)… (1 being least easy/5 being most easy)

1. Easy to use? (1-2-3-4-5)
2. Something you would be willing to do again if it helped your future care? (1-2-3-4-5)

### Feasibility

For each tool (mFI/CFS/Optimal Preoperative Assessment guideline) we will measure:

1. Time to administer
2. Number of missing items (based on typical practice for lab tests and diagnostics-no extra lab tests will be ordered as part of this study protocol)

## Abbreviations

ACS: American College of Surgeons
AD8: Ascertain Dementia 8-item questionnaire
AGS: American Geriatrics Society
CAGE: Cut down/Annoyed/Guilty/Eye-opener (screen for alcoholism)
CFS: Clinical frailty scale
CRA: Clinical research assistant
DFS: Disability free survival
LOS: Length of stay
mFI: Modified frailty index
PROM: Patient-reported outcome measure
REB: Research ethics board
rFPR: Relative false positive rate
ROC: Receiver operator curve
rTPR: Relative true positive rate
WHODAS: World health organization disability assessment schedule
